# Effects of resourcefulness on internet game addiction among college students: The mediating role of anxiety and the moderating role of gender

**DOI:** 10.3389/fpubh.2023.986550

**Published:** 2023-02-13

**Authors:** Yan Zhang, Yun-Ling Zhong, Jing Luo, Jin-Long He, Cen Lin, Jaclene A. Zauszniewski, Jin-Hui Zhou, Ying Chen, Chun-Yan Wu, Shu-Rui Wang, Zheng-Huan Li, Jing Tang, Wan-Ning Li, Jing Wu, Jia-Ming Luo

**Affiliations:** ^1^School of Psychiatry, North Sichuan Medical College, Nanchong, Sichuan, China; ^2^Frances Payne Bolton School of Nursing, Case Western Reserve University, Cleveland, OH, United States; ^3^Mental Health Center, Southwest Petroleum University, Nanchong, Sichuan, China; ^4^Department of Neurology, Affiliated Hospital of North Sichuan Medical College, Nanchong, Sichuan, China

**Keywords:** resourcefulness, anxiety, internet game addiction, gender, college students

## Abstract

**Introduction:**

The mechanism of internet game addiction is unclear. Whether anxiety mediates between resourcefulness and internet game addiction and whether gender affect its mediation role have not been studied previously.

**Methods:**

A total of 4,889 college students from a college in southwest China were included in this study to complete the investigation, in which three questionnaires were used for evaluation.

**Results:**

Pearson's correlation analysis indicated a remarkable negative correlation between resourcefulness with internet game addiction and anxiety, as well as a significant positive correlation between anxiety and this addiction. The structural equation model confirmed the mediation role of anxiety. The multi-group analysis confirmed the moderating role of gender in the mediation model.

**Discussion:**

These findings have advanced the results of existing studies, indicating the buffering effect of resourcefulness on internet game addiction and revealing the potential mechanism of this relationship.

## Introduction

With the popularity of the internet comes potential problems. There is evidence that some college students are using the internet irrationally and are even addicted to online games (1). Internet game addiction refers to “the persistent and repeated use of the internet to engage in games that result in impairment of daily life, and the tendency to isolate oneself socially” (2, 3). This phenomenon is prevalent around the world, with one meta-analysis showing a global prevalence of 3.05% for internet game addiction (4). Studies have also reported that the prevalence of internet game addiction is 11% in the Chinese college student population (5). Internet game addiction has been officially listed under ICD-11 (the International Classification of Diseases 11th Revision) and has become a public health issue that can have a range of negative effects on college students. Internet game addiction could lead to sleep insufficiency, depression, academic difficulties, and poor creativity and productivity. It impairs the physiological, psychological and social functioning of college students (6).

“Resourcefulness is a combination of an individual's ability to carry out everyday tasks independently and the ability to seek help from outside sources when appropriate” (7, 8). It typically comprises of personal and social dimensions. “Personal resourcefulness” is the ability of an individual to maintain daily life independently, for example by using personal effort or internal resources to achieve goals in the face of potentially adverse stressful situations and stimuli (7). “Social resourcefulness” is the ability to seek help from formal or informal sources when an individual is unable to deal with a problem on his or her own (8). Although there are no previous theories that directly discuss resourcefulness and internet game addiction, Zauszniewski's *Theory of Resourcefulness and Quality of Life*^©^ may provide support. This theory suggests that resourcefulness has a direct impact on a person's quality of life and that internet game addiction can be considered an indicator of quality of life (8–10). Although this is the first study of personal and social resourcefulness and internet game addiction, there have been empirical studies that have examined the relationship between closely related construct and addictive behavior. For example, two studies have examined the relationship between “learned resourcefulness” and addictive behavior. In these studies, “learned resourcefulness” was operationalized through self-control, which did not consider seeking help from others as a characteristic of resourcefulness (8). Accordingly, Kennett et al. reported that persons with greater “learned resourcefulness” were better able to change their alcohol-drinking and smoking habits (11). Bulut and Zeren found that Internet addiction could be predicted by “learned resourcefulness” (12). In terms of social resourcefulness, Rapp et al. reported that social resourcefulness leads to more social support (13). According to the main effect model of social support, social support affects internet addiction (14), and this relationship has similarly been tested in empirical studies (15–17). Taken together, *the Theory of Resourcefulness and Quality of Life*^©^, the main effect model of social support (14), and related empirical studies all suggest that resourcefulness may be an important variable in predicting internet game addiction.

**Figure 1:**
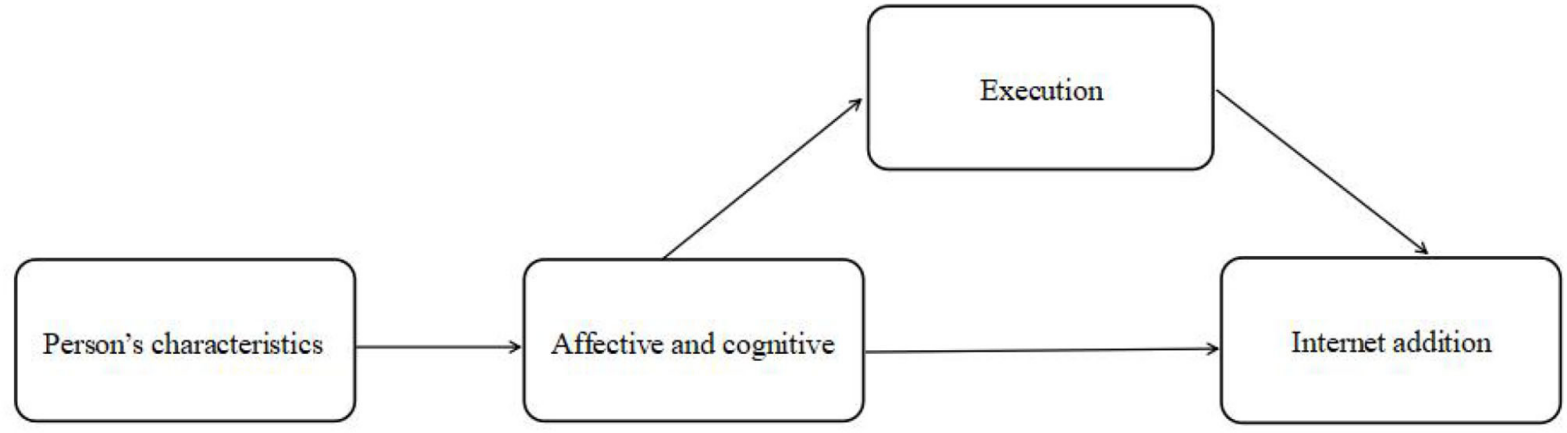
I-PACE model for internet addiction.

The mechanism of the influence of resourcefulness on internet game addiction is unclear, and whether anxiety plays a mediating role has not been studied before. The Interaction of Person-Affect-Cognition-Execution (I-PACE) model was chosen as the framework for this study. This model emphasizes that addictive behaviors are the consequence of interactions between predisposing factors (Person's characteristics), mediators (affective and cognitive responses) and execution (Figure 1) (18). In this study, resourcefulness is a relatively stable personal characteristic, and it refers to the ability to learn (19, 20). Therefore, resourcefulness is considered a predisposing variable. Anxiety is included as a mediating variable. Internet game addiction is considered a dependent variable. Specifically, individuals who are not good at using personal and social resources may face real-world difficulties and a worse emotional state (21), and they may develop internet addiction in regulating emotions (22). Regarding personal resourcefulness, studies have shown that anxiety and depression mediate between personal resourcefulness and life satisfaction (23). As for social dimension, social resourcefulness will bring more social support (13). Social support may indirectly affects human behaviors (e.g., internet addiction and suicide) *via* emotional state (14). This is supported by a related empirical study that suggests depression mediates between social support and suicidal ideation (24). Based on the reasoning above, the relationship between resourcefulness and internet gaming addiction should consider a psychological model that includes anxiety.

*The Theory of Resourcefulness and Quality of Life*^©^ has four components, i.e., antecedent situational factors (internal demographic characteristics and external environmental factors), process regulators (perceptive, cognitive, affective, motivational, and volitional), resourcefulness (personal resourcefulness and social resourcefulness), and life quality indicators (8). Resourcefulness can directly influence quality of life, and both anxiety and internet game addiction were conceptualized as quality of life indicators in this study. Zauszniewski et al. mentioned in a previous study resourcefulness influenced anxiety (anxiety as a quality of life indicator) and vice versa (anxiety as a process regulator) (25), and this study focused on the former. Previous studies in caregivers of people with dementia (26), older adults (27), adolescents (28), and pregnant women (29) have found that resourcefulness negatively predicts anxiety, depression. And, anxiety is a major risk factor for Internet addiction, with both cross-sectional and longitudinal studies providing empirical evidence for the relationship (30, 31). One study found that cognition mediates the relationship between “learned resourcefulness” and adaptive functioning (32). Another study among college students showed that self-control influenced social anxiety, which in turn led to negative emotions and internet addiction (33). Thus, anxiety may play a mediating role.

In evaluating the factors associated with online game addiction, researchers consider demographic factors. Gender differences in addictive behaviors have been studied and are important evidence in understanding online game addiction. Some studies have noted that compared with females, males have poorer self-control ability and are bad at seeking social support (34), so they may have a higher risk of developing negative emotions and online games (35, 36). However, the opposite finding still exists, with females scoring higher than males in online gaming addiction (37). In addition, there are studies that do not find an association between gender and online addiction, possibly due to factors such as the popularity of the internet and the purpose of use (38). In summary, although no unanimous conclusion has been reached regarding the effect of gender on self-control, anxiety, and internet game addiction, the greater extent suggests that resourcefulness, anxiety, and internet game addiction may change with gender differences, and the mediating role of anxiety may also vary.

In summary, this study proposes the hypotheses that: resourcefulness has a predictive effect on internet game addiction, and anxiety is one of the mediating factor. This study also hypothesize that gender moderates the mediating effect, i.e., the mediating role of anxiety vary between male and female groups.

## Methods

### Participants

This is a cross-sectional survey study conducted in Southwest China in October 2022. A convenient sampling method was used to administer a questionnaire to students of clinical medicine, nursing science, medical imagology, clinical medicine of traditional Chinese medicine and western medicine, anesthesiology, pharmacy, preventive medicine, stomatology, optometric medicine and ophthalmology, medical laboratory technology, midwifery, management science, foreign language and culture, biomedical engineering, and athletic rehabilitation. Inclusion criteria: college students at school; signed an informed consent form and voluntarily joined the study. Exclusion criteria: those with severe mental disorders that prevented them from cooperating with the survey; those who did not wish to join the study. The researcher distributed the questionnaire star QR code or link to the subjects through social media platforms, and the participants voluntarily filled in the questionnaire after reading the informed consent form. A total of 5,523 questionnaires were distributed in this study and 4,899 valid questionnaires were collected, with a response rate of 88.7%. Among them, 1,758 (35.9%) were male students and 3,141 (64.1%) were female students. The number of freshmen to 5th-year students were: 2,003 (40.9%), 970 (19.8%), 1,219 (24.9%), 574 (11.7%), and 133 (2.7%), respectively. The age of the subjects ranged from 16 to 25 years, with an average age of 19.54 ± 1.46 years. The Ethics Committee of North Sichuan Medical College confirmed that the present study adhered to ethical principles.

### Measures

#### Demographics

Several demographic variables were collected for this study: age, grade, major, gender (1 = boy, 2 = girl), place of residence (1 = urban, 2 = rural), and whether or not the child was an only child (1 = parent with one child, 2 = parent with more than one child).

#### Resourcefulness

Resourcefulness was measured using the Chinese version of the Resourcefulness Scale^©^, which was the translated version by Lai and Wang et al. (39, 40) of RS^©^ developed by Zauszniewski et al. (7). The C-RS^©^ consists of two dimensions of personal resourcefulness (16 items) and social resourcefulness (12 items). The Likert 6-point scale is used, with higher scores indicating higher levels of resourcefulness. The Cronbach's alpha coefficient for the Chinese version of the scale was 0.898, and those for the dimensions were 0.875 and 0.797.

#### Anxiety

SAS was designed by Zung (41). This study used the Chinese translation of the SAS to measure anxiety (42). The scale has 20 items and is rated on a 4-point scale, with higher scores indicating higher levels of anxiety. The Cronbach's alpha coefficient for the Chinese translated version of the SAS in this study was 0.844.

#### Internet game addiction

Nine diagnostic criteria for internet game disorder were put forward in *The Diagnostic and Statistical Manual of Mental Disorders* (DSM-5) (43, 44). On this basis, Pontes et al. developed Nine-Item Internet Gaming Disorder Scale (IGDS9-SF) (45). This study used the revised IGDS9-SF to measure internet game addiction (46, 47). The scale has nine question items and is scored on a five-point scale, with higher scores associated with higher levels of internet game addiction. The Cronbach's alpha coefficient for the revised IGDS9-SF in this study was 0.902.

#### Quality control

The research design phase involved forming a research team with psychiatry and psychology professionals to discuss the research design, select survey instruments and determine survey procedures and methods.

The data collection phase was conducted in a classroom setting, with the researcher distributing the QR code of wjx.cn through social media platforms and subjects scanning the code to access the web-based questionnaire system. Before subjects completed the survey, they were required to read the purpose of the survey, the method of completion, informed consent, and informed that the survey was anonymous. After consenting, subjects voluntarily participated in the online questionnaire to ensure that the study data were authentic and valid.

In the stage of data completion and analysis, researchers exported the data from wjx.cn and imported them into the SPSS24.0 software. The data analysts were trained uniformly, thus ensuring the accuracy of data.

#### Data analysis

The data were analyzed using SPSS 24.0. Cronbach's alpha coefficient represented the reliability of the questionnaire. Harman's one-factor test was performed to assess the common method bias. Pearson's correlation analysis was conducted to analyze the correlation among variables. A structural equation model was established by Amos 24.0. The mediating effect was tested by the percentile Bootstrap method for bias calibration; the moderating effect was measured by multi-group analysis. Due to the impact of sample size on chi-square values, the model's fitting result was evaluated by comparative fit index (CFI), Tucker-Lewis index (TLI), root mean square error of approximation (RMSEA), and standardized root mean square residual (SRMR). According to the researcher's recommendation, CFI and TLI > 0.90 and RMSEA and SRMR < 0.08 were used as the criteria to evaluate the goodness of fit data of the model.

## Results

### Common method biases analyses

To control the possible common method bias, this study used questionnaires with reverse scoring and different rating scales. The Harman's One-factor Test was used to test for common method bias. The results showed that nine factors with a characteristic root >1. The first factor explained 17.53% of the variation, which was much less than the critical value of 40% (48). Therefore, there was no significant common method bias in this study.

### Correlation analysis of variables

Pearson's correlation analysis was conducted on resourcefulness, anxiety and internet game addiction (Table 1). The results showed that resourcefulness was significantly and negatively correlated with both internet game addiction (*r* = −0.150, *P* < 0.001) and anxiety (*r* = −0.243, *P* < 0.001). Besides, anxiety was significantly and positively correlated with internet game addiction (*r* = 0.322, *P* < 0.001). This provided initial support for further testing.

**Table 1:** Correlation analysis of variables.

**Variable**	***M***	***SD***	**1**	**2**	**3**	**4**
1 Gender^a^	0.64	0.48	1			
2 Resourcefulness	3.39	0.53	−0.025	1		
3 Anxiety	1.64	0.36	−0.058^***^	−0.243^***^	1	
4 Internet game addiction	1.74	0.66	−0.365^***^	−0.150^***^	0.322^***^	1

a Gender is a dummy variable (males = 0; females = 1) and the mean value represents the proportion of females.

*** *p* < 0.001.

### Analysis of the mediating effect of anxiety

**Figure 2:**
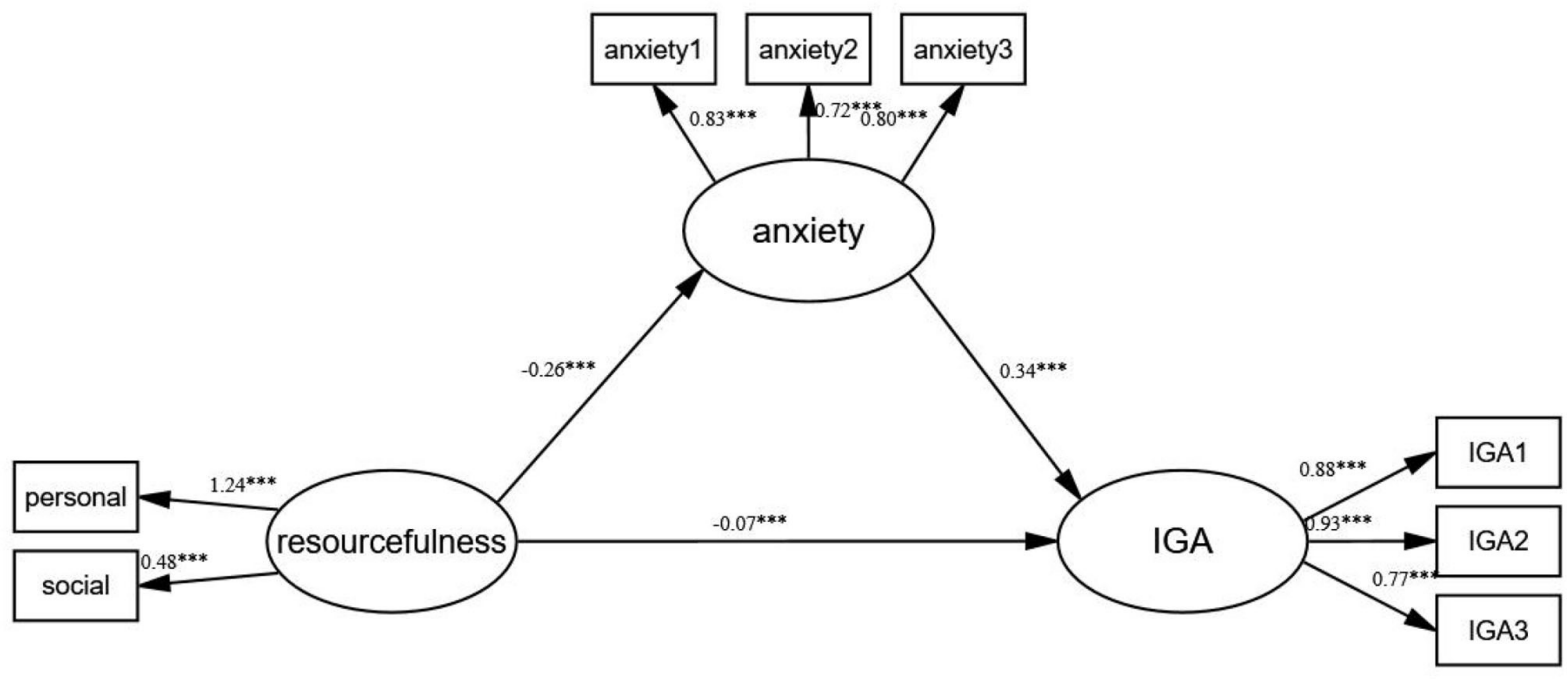
Model for the mediating effect of anxiety. ***p < 0.001.

To address the problem of latent variables containing multiple observing indexes, this study used a completely random packing method to pack resourcefulness, anxiety, and internet game addiction into 2, 3, and 3 indexes, respectively (49). A structural equation model was developed with resourcefulness as the independent variable, internet game addiction as the dependent variable, and anxiety as the mediating variable (Figure 2). The results showed that the model fitted well, with all fit indices within a reasonable range (χ^2^/*df* = 16.87, CFI = 0.98, TLI = 0.97, SRMR = 0.03, RMSEA = 0.05). Based on these fitting results, the non-parametric percentile Bootstrap method for bias calibration was used to test the mediating effect and evaluate the confidence interval (CI), during which the sampling was repeated 5,000 times. Results showed that resourcefulness negatively predicted internet game addiction (−0.16, *P* < 0.001) and anxiety partially mediated the process (β = −0.09, 95% CI = −0.1 to −0.07), accounting for 56.25% of the total effect. The 95% CI did not include 0, which verified the mediating effect of anxiety (Table 2).

**Table 2:** The mediating effect of anxiety.

**Effect**	**β**	***SE***	**95% CI**	***P***
Total effect	−0.16	0.01	[−0.18, −0.14]	<0.001
Direct effect	−0.07	0.01	[−0.09, −0.05]	<0.001
Indirect effect	−0.09	0.008	[−0.1, −0.07]	<0.001

### Analysis of the moderating effect

Multi-group analysis was conducted to identify whether the path coefficients differ significantly between females and males. Unconstrained model M1, structural weight model M2, and structural residual model M3 were developed, respectively. As displayed in Table 3, the results showed that the constrained models (M2 and M3) were significantly different from the unconstrained model (M1; *P* < 0.001), suggesting significant gender difference. Further comparing the difference in the coefficients, we found gender played a role in the following pathways.

**Table 3:** The multi-group analysis results.

**Model**	**χ^2^**	***Df***	**CFI**	**RMSEA**	**Δχ^2^**	**Δ*df***	***P***
M_1_	320.79	34	0.98	0.04	-	-	-
M_2_	350.71	39	0.98	0.04	29.93	5	<0.001
M_3_	387.10	42	0.98	0.04	66.31	8	<0.001

From resourcefulness to anxiety, the path coefficients were −0.24 (*p* < 0.001) and −0.29 (*p* < 0.001) for the male and female groups, respectively. The absolute value of critical ratios for differences between parameters was 4.66 (>1.96), significantly different at the 0.05 level. Resourcefulness could reduce anxiety more effectively in females than in males.

From anxiety to internet game addiction, the path coefficients were 0.40 (*p* < 0.001) and 0.30 (*p* < 0.001) for the male and female groups, respectively. The critical ratios for differences between parameters showed an absolute value of 3.08 (>1.96), significantly different at the 0.05 level. The predictive effect of anxiety on internet game addiction was stronger for males compared to females.

## Discussion

This study examined the relationship between resourcefulness, anxiety and internet game addiction among Chinese college students. This is the first time that the potential influence mechanism of resourcefulness on college students' addiction to online games has been discussed. This study extended the results of previous studies on internet game addiction and its internal psychological mechanism. Also, it provided an empirical reference for preventing and intervening such addiction among college students.

### The relationship between resourcefulness and internet game addiction

The present study found that resourcefulness significantly and negatively predicted internet game addiction, supporting our hypothesis. That is, having resourcefulness is effective in reducing the risk of internet game addiction, which is similar to previous findings. For example, Bulut and Zeren found that learned resourcefulness reduced online addiction (12). One possible explanation for this is that resourcefulness is similar to a resilient defense that serves as a protective buffer. Individuals who possess resourcefulness are more resilient, have better coordination and adaptability, and are less likely to become addicted to the Internet (50). Another possible explanation is that individuals who lack resourcefulness have a poor level of self-control. Their behaviors are mainly controlled by immediate gratification and short-term goals. They tend to seek immediate pleasure and rewards in online games (51, 52).

### The mediating effect of anxiety

The main finding of the present study is that anxiety partially mediated the relationship between resourcefulness and internet game addiction, supporting our hypothesis, which, to our knowledge, has not been directly examined. Thus, our study, to a certain extent, fills the gap in exploring the influence mechanism of resourcefulness on internet game addiction. The mediating role of anxiety can be explained by the I-PACE model, which assumes that the occurrence and development of addictive behaviors result from predisposing variables, affective and cognitive responses, and executive (18). In this study, resourcefulness serves as the independent variable that influences internet game addiction through anxiety. A person's inability to self-regulate and lack of social support may lead to real-world difficulties and worsen the individual's emotional state (21). They may seek satisfaction in the virtual online world when regulating their emotions and develop a dependency (53). Overall, individuals who lack resourcefulness are prone to develop more anxiety. In this instance, they often vent their anxiety by playing online games, which will easily result in addiction to these games. Conversely, individuals who have resourcefulness may have less anxiety and are less vulnerable to internet game addiction.

### The moderating effect of gender

Another important finding of this study was that the effect of resourcefulness on anxiety was more obvious in females than in males. The “protective-responsiveness model” states that the effect of a protective factor is stronger when another risk factor is high (54). Resourcefulness is like an elastic line of defense, performing protective and buffer functions. Studies have shown that females are more emotionally sensitive and may be at higher risk for anxiety (55–57). Thus, the effect of resourcefulness on anxiety is more obvious in females.

In addition, the effect of anxiety on internet game addiction was stronger in males than in females. One explanation for this result could be that social norms and expectations of males play a reinforcing role. According to social role theory, males and females play different roles in society. In the Chinese cultural context, males are portrayed as independent, strong and successful, while females are portrayed as docile, affectionate and easy to ask for help. They may behave according to their own understanding of their roles and society's expectations of them (58). Among people who feel anxious, females may be more inclined to express themselves, shop, and seek interpersonal support, influenced by social roles and norms. Whereas, males may be more inclined to work through it alone, online gaming would be an option. Another possible explanation is that neural mechanisms make the effect of anxiety on internet game addiction more obvious in males. According to the gender difference in neural mechanism, gaming cues will induce a stronger desire in males, so they can easily experience something new through online games. In the meantime, the competitive structure in these games is more attractive to males, which activates the area of the brain related to awards (59, 60).

### Limitations and implications

The findings confirm that resourcefulness can negatively predict internet game addiction, with anxiety playing the meditating role. Besides, gender moderates this mediating effect. These findings to a certain extent enrich the existing research on internet game addiction and its underlying psychological mechanisms. The present study suggests that we should pay more attention to college students' state in playing online games and improve their mental health by enhancing resourcefulness and balancing emotions. Further, we should make greater efforts to evaluate and educate females about their resourcefulness level and males about their emotion management.

However, there are some limitations in the present study, which indicates the direction of future research. First, despite the large sample size, more representative samples shall be selected from a wider range. Besides, the large sample size may affect the significance of correlation analysis, as well as mediation, and moderating effects. Second, we cannot conduct causal inference since this is a cross-sectional study. In this respect, a longitudinal tracking study shall be conducted to make a more detailed exploration. Third, there may be some other potential variables that affect the results. For instance, depression may also mediate the association between resourcefulness and internet game addiction. So we shall explore other variables further to provide more references for the intervention and treatment of individuals' internet game addiction.

## Conclusion

Resourcefulness can negatively predict the internet game addiction of individuals in a significant way.

Resourcefulness can indirectly predict the internet game addiction of individuals through anxiety.

Anxiety mediates the relationship of resourcefulness and internet game addiction differently between males and females.

## Data availability statement

The raw data supporting the conclusions of this article will be made available by the authors, without undue reservation.

## Ethics statement

The studies involving human participants were reviewed and approved by the Ethics Committee of North Sichuan Medical College. The patients/participants or patients/participants' legal guardians/next of kin provided their written informed consent to participate in this study.

## Author contributions

YZ was involved in the study design and the composing of this manuscript. J-ML and Y-LZ provided the subject of this study and critically revised this manuscript. JL, J-LH, and CL searched and reviewed the references. JZ provided Resourcefulness Scale^©^, constructive comments, and revised the manuscript. J-HZ completed the data analysis. YC and JW modified this manuscript. C-YW, S-RW, Z-HL, JT, and W-NL collected the data. All authors contributed to this manuscript and approved the submitted version.
